# Pitx2 Differentially Regulates the Distinct Phases of Myogenic Program and Delineates Satellite Cell Lineages During Muscle Development

**DOI:** 10.3389/fcell.2022.940622

**Published:** 2022-07-06

**Authors:** Felícitas Ramírez de Acuña, Francisco Hernandez-Torres, Lara Rodriguez-Outeiriño, Jorge N. Dominguez, Lidia Matias-Valiente, Cristina Sanchez-Fernandez, Diego Franco, Amelia E. Aranega

**Affiliations:** ^1^ Cardiac and Skeletal Myogenesis Group, Department of Experimental Biology, University of Jaen, Jaén, Spain; ^2^ Cardiac and Skeletal Myogenesis Group, MEDINA Foundation, Center for Excellence in Research of Innovative Medicines in Andalusia, Granada, Spain; ^3^ Department of Biochemistry, Molecular Biology III and Immunology, School of Medicine, University of Granada, Granada, Spain

**Keywords:** Pitx2, myogenic precursors, satellite cells, myogenesis, somites

## Abstract

The knowledge of the molecular mechanisms that regulate embryonic myogenesis from early myogenic progenitors to myoblasts, as well as the emergence of adult satellite stem cells (SCs) during development, are key concepts to understanding the genesis and regenerative abilities of the skeletal muscle. Several previous pieces of evidence have revealed that the transcription factor *Pitx2* might be a player within the molecular pathways controlling somite-derived muscle progenitors’ fate and SC behavior. However, the role exerted by *Pitx2* in the progression from myogenic progenitors to myoblasts including SC precursors remains unsolved. Here, we show that *Pitx2* inactivation in uncommitted early myogenic precursors diminished cell proliferation and migration leading to muscle hypotrophy and a low number of SCs with decreased myogenic differentiation potential. However, the loss of *Pitx2* in committed myogenic precursors gave rise to normal muscles with standard amounts of SCs exhibiting high levels of *Pax7* expression. This SC population includes few MYF5^+^ SC-primed but increased amount of less proliferative miR-106b^+^cells, and display myogenic differentiation defects failing to undergo proper muscle regeneration. Overall our results demonstrate that *Pitx2* is required in uncommitted myogenic progenitors but it is dispensable in committed precursors for proper myogenesis and reveal a role for this transcription factor in the generation of diverse SC subpopulations.

## Introduction

In vertebrates, all muscles in the trunk and limbs derive from myogenic precursor cells (MPCs) expressing *Pax3*, which are present in somites, transient structures that form pairwise on either side of the neural tube. As the somite matures, myogenic progenitor cells become confined to the dorso-lateral part of the somite: the dermomyotome (DMT) (Buckingham, 2017; Chal and Pourquié, 2017; Hernandez-Torres et al., 2017). Soon after, primary myogenesis start and sequential waves of PAX3^+^ and PAX3^+^/PAX7^+^ MPCs in the DMT undergo an epithelial-to-mesenchymal transition, migrate to its ventral surface and progressively express *Myf5*, *Myod1*, and *Myf6* giving rise to DESMIN + myoblasts that form the myotome (MT). Myogenic progenitors located in the dorsomedial part of the MT configure the epaxial myotome which form the back muscles, while the ventrolateral region or hypaxial MT gives rise to body wall and intercostal muscles. However, PAX3^+^ positive MPCs from the hypaxial DM migrate before differentiating into hypaxial myotome and originate the muscles of the limb, tongue and diaphragm. These mesenchymal cells remain undifferentiated and mesenchymal during the migration (Buckingham et al., 2003; Scaal and Christ, 2004; Vasyutina and Birchmeier, 2006; Bentzinger et al., 2012). Secondary myogenesis then proceeds through diverse waves of differentiation, initially a group of embryonic myoblasts form the primary muscle fibers representing a scaffold for the secondary myofibers formed by the fusion of fetal myoblasts around E14.0 in mice (Buckingham et al., 2003; Hernandez-Torres et al., 2017). Therefore, developmental myogenesis comprises distinct but overlapping steps involving different types of myogenic cells: early myogenic precursors, embryonic myoblasts and fetal myoblasts (Rodriguez-Outeiriño et al., 2021). An important issue in the field is to better understand how the molecular machinery regulating myogenesis is acting on those diverse myogenic cell populations during skeletal muscle development.

Moreover, it is interesting to highlight that, although it is well accepted that adult SCs arise from Pax3/Pax7-expressing cells in the DMT (Gros et al., 2005; Relaix et al., 2005; Schienda et al., 2006; Hutcheson et al., 2009; Lepper et al., 2009) the embryonic origin of SCs is still an open issue (Gopalakrishnan et al., 2015; Lescroart et al., 2015; Tzahor, 2015). In this sense, it is interesting to note that the genetic and cellular mechanisms by which SC lineage is maintained as myogenic progenitors during further embryonic and fetal development while progenitors residing beside actively differentiate remain elusive. Since Pax3 and Pax7 genes are expressed in progenitors for all muscle precursor subtypes, it has been proposed that another cell intrinsic and extrinsic mechanism could be cooperating with Pax3 and Pax7 proteins to specify SC fate (Murphy and Kardon, 2011; Tierney and Sacco, 2016). The transcription factor Pitx2 is expressed in MT and all developing muscles of the trunk, including the limb muscles (Kitamura et al., 1999; Shih et al., 2007a). Several previous works have provided some evidence about the initial role of Pitx2 at the onset of myogenesis acting downstream of Pax3 and regulating Myod1 expression (Shih et al., 2007b; L’honoré et al., 2010). Besides, we and others have revealed that Pitx2 is also related to cell proliferation in myogenic cells and somite derivatives by controlling the expression of cell cycle genes (Kioussi et al., 2002; Martínez-Fernandez et al., 2006; Abu-Elmagd et al., 2010). Moreover, our previous works have shown that Pitx2 is present in a subset of adult SCs and positively regulates SC differentiation by activating a PITX2-miR-106b/miR-503/miR-23b/miR-15b pathway indicating a role of this transcription factor in SC biology (Lozano-Velasco et al., 2015; Vallejo et al., 2018). However, how Pitx2 is acting in different myogenic progenitors’ populations during myogenic progression, including SC precursors, remains to be explored.

In this study we show that *in vivo* inactivation of Pitx2 in early predetermined uncommitted MPCs by using the Pax3^Cre/Wt^ deleter line leads to defective cell proliferation and migration of MPCs generating muscle hypotrophy. We also found a lower number of SCs in the muscles of Pax3^Cre/Wt^/Pitx2^−/−^ conditional mutant mice with decreased capabilities to form myotubes *in vitro* and deficient regenerative abilities *in vivo*. Interestingly, Pitx2 inactivation in myogenic committed progenitors, by using Myf5^Cre/Wt^ deleter line, give rise to apparent normal muscles that contain SCs expressing high levels of Pax7 that contain a reduced number of MYF5^+^-primed but increased amount of less proliferative miR-106b^+^ cells displaying deficient SC differentiation potential and severe defects on muscle regeneration. These results reveal unknown functions of Pitx2 in uncommitted and committed myogenic progenitors and provide evidence for a role of this transcription factor defining SC subpopulations.

## Materials and Methods

### Generation of Conditional Tissue-Specific Null Mutant Mice

Animal procedures were approved by the University of Jaen Ethics Committee, and it was conducted according to the national and European community guidelines regulations for animal care and handling. All mice were maintained inside a barrier facility where food and water were administered *ad libitum*. All experiments were performed in accordance with University of Jaen regulations for animal care and handling (16/07/2019/130).

B6; 129-Pax3^tm1(cre)Joe/J^ (ref. 005549) transgenic mice were purchased from The Jackson Laboratory. Myf5^Cre/Wt^ were kindly supplied by Victor Luis Ruiz Pérez, (Instituto de Investigaciones Biomédicas de Madrid). Generation of conditional Pax3Cre and Myf5Cre mutants was performed by intercrossing hemizygous Cre deleter with homozygous Pitx2^Floxed/Floxed^ mice which were kindly supplied by Marina Campione (Pathophysiology of Striated Muscle Group, Universitá degli Studi di Padova). Those Pitx2^Floxed/Floxed^ mice have been widely used to generate conditional mutant Pitx2 that lose activity of all 3 isoforms as a consequence of the homeodomain deletion (Tessari et al., 2008; Chinchilla et al., 2011; Ammirabile et al., 2012). Double heterozygous were selected by PCR and subsequently crossed with homozygous Pitx2floxed mice, respectively. The mice were PCR screened with Pitx2-floxed and Cre-specific primers (Table 1).

**TABLE 1:** Genotyping sequences.

Primer	Sequence	
Pitx2ln4_f01	GGT​GGG​GGT​GTC​TGT​AAA​AC	Pitx2^Flox/Wt^
Pitx2ln5_r01	CAA​GCC​TTG​CGT​GTT​TCT​G	Pitx2^Flox/Wt^
oIMR6977	CTG​CAC​TCA​AGG​GAC​TCC​TC	Pax3^Cre/Wt^
oIMR6978	GTG​AAG​GCG​AGA​CGA​AAA​AG	Pax3Cre^Cre/Wt^
oIMR9074	AGG​CAA​ATT​TTG​GTG​TAC​GG	Pax3Cre^Cre/Wt^
AF-Cre1	CGG​TCG​ATG​CAA​CGA​GTG​ATG​AGG	Myf5^Cre/Wt^
AF-Cre2	CCA​GAG​ACG​GAA​ATC​CAT​CGC​TCG	Myf5^Cre/Wt^

### Whole-Mount *In Situ* Hybridization

Mouse embryos from E10.5 and E12.5 were fixed and stored in 4% PFA at 4°C. Complementary RNA digoxigenin-labelled probes of Pax3, Myod1 and MyoG were generated using standard protocols. The embryos were washed in PBT (PBS x10, 0,1% Tween^®^ 20) and MetOH to be dehydrated before being bleached (6% H2O_2_/MetOH) in darkness. After being rehydrated with PBT, the embryos were treated with Proteinase K and postfix (0,2% glutaraldehyde 4% PFA). Prehybridization washes (50% Formamide, 25% SSC x20 pH4.5, 2% SDS, 2%nBBR, 0.025% yeast tRNA, 1% Heparin) and hybridization were carried out at 70°C with following washes (50% Formamide, 20% SSC x20 pH4.5, 2% SDS) at the same temperature. The staining (0,15% NBT, 0,2% BCIP on NTMT) was performed at RT.

### Fetal Myoblasts and SCs and Cultures

Myoblast fusion experiments with primary cells from hindlimb muscle were carried out as described (Schwander et al., 2003). For fusion experiments, equal numbers of wild-type and β1-deficient cells were mixed. The number of myotubes was determined by counting MF20 positive myotubes with two or more nuclei on ten fields. The mean and standard deviation were calculated.

For SC (SC) isolation, hindlimb skeletal muscles from 2-3-month-old male mice were isolated by enzymatic/mechanical dissociation followed by magnetic-activated cell sorting (MACS) according to the manufacturer’s protocol (Miltenyi Biotec). Briefly, muscles were minced and digested for 1 h at 37°C in an enzymatic solution containing DMEM:F12 (1:1) and supplemented with 1 mg/ml collagenase-type D and 4% Trypsin solution. Afterwards, a mechanical dissociation was performed through 14G and 18G needle up and down. The supernatants were collected and filtered through 100 and 40 μm cell strainers. Suspension cells were pelleted (300 g for 5 min) and resuspended with 1 ml of PBS for incubation with Red Blood Cells Lysis Solution (130-094-183, Miltenyi Biotec) for 2 min. Incubation with magnetic labelling was performed for 15 min at 4°C of 1∶5 dilution according MicroBeads SC Isolation Kit (negative selection with SC Isolation Kit-130-104-268, and positive selection with Anti- Integrin α-7 MicroBeads-130-104-261, Miltenyi Biotec). Purified mouse SCs were seeded on 0.1% gelatin coated dishes and cultured in mouse growth medium, containing DMEM GlutaMAXTM (Life technologies) and DMEM:F12 (Lonza) 50:50 supplemented with 20% FBS (Sigma), 2% Ultroser^®^ G (Pall), 3% Penicillin/Streptomycin (P/S) and incubated at 37°C, 5% CO_2_. For myoblast differentiation, when 70–80% confluence was reached, the medium was changed, decreasing to 2% FBS.

### Histology, Immunohistochemistry Analysis in Mouse Embryos and Adult Muscles Including Cardiotoxin Injury

Tibialis anterioris muscles were collected and frozen either in liquid nitrogen-cooled isopentane (for sectioning) or in liquid nitrogen (for total RNA isolation). For Hematoxylin-eosin staining, 10 μm thick cryosections samples were pre-fixed with 4% PFA for 10 min at RT and incubated for 10 min with Mayer’s Hematoxylin Solution. Afterward, the samples were incubated in activated Eosin Y Solution 0.5% for 10 min, dehydrated and mounted in DPX. For immunohistochemical staining in, 10 μm thick sections were used. Muscle cryosection samples were pre-fixed with 4% PFA for 10 min at RT. For PAX7 immunostaining, epitopes were unmasked in citrate buffer (10 mM Sodium Citrate, 0.05% Tween-20, pH 6.0) in a pre-heat water bath 30 min at 95°C. Afterwards, all samples were incubated in TBSA-BSAT (10 mM Tris, 0.9% NaCl, 0.02% sodium azide, 2% BSA and 0.1% Triton X-100) at RT for 30 min. All primary antibodies (Supplementary Table S1) were diluted to 5 μg/μl into TBSA-BSA and incubated overnight at 4°C in a humidified chamber. Alexa-conjugated secondary antibodies (Supplementary Table S1) were diluted 1/200 in TBSA-BSAT and applied to the samples for 2 h at RT. Finally, sections were incubated with DAPI in PBS 1/2000 for 15 min at RT and mounted with Hydromount (National Diagnosis. HS-106).

For each *in vivo* experiment, 4-month-old male mice were anesthetized by using isoflurane, 2%–5% inhaled, to a surgical plane of anesthesia. For cardiotoxin (CTX) injury, tibialis anterior (TA) muscles were injected with 50 µL of 10 μM CTX/PBS using a 25G needle. Then, the animals were sacrificed and TA muscles were collected at 3, 7 and 15 days after cardiotoxin injection. For immunohistochemical staining in, 10 μm thick sections were used and processed as described above. For tissue samples from paraffin, the slides were heated at 60°C during 10 min and dewax in xylene washes and rehydrated in diminishing alcohol washes before being blocked with TBSA solution.

Embryos of E10.5, E12.5 and E14.5 were fixed for 20, 40 and 60 min respectively at room temperature and then dehydrated in an ascending series of ethanol (70°, 80°, 90°, Absolute). They were then placed in a solution of butyric alcohol, butyric-paraffin wax (1:1) and three consecutive paraffin wax steps before embedding embryos in paraffin blocks. Histological and immunohistochemical analyses were performed as described above. Primary and secondary antibodies are listed in Supplementary Table S1.

Cultured cells were fixed with 4% PFA 20 min and permeabilized with PBS, 0.25% Triton X-100 and 50 mM NH_4_Cl 10 min at RT. Then, the samples were blocked with 0.2% gelatin/PBS (G1393) 20 min at RT. Primary antibodies (Supplementary Table S1) were diluted to 5 μg/μl in blocking solution and applied overnight at 4°C. Alexa-conjugated secondary antibodies were added at dilution 1/300 in blocking solution for 30 min at RT. Finally, samples were incubated with DAPI in PBS 1/2000 for 15 min at RT and mounted with Hydromount (National Diagnosis. HS-106).

To analyze the size of skeletal muscle fibers, we measured the fiber cross sectional area as described (Moresi et al., 2009) using ImageJ software on adult and neonatal TA slices.

### MicroRNA *In-Situ* Hybridization

FISH for microRNA detection was performed in cryosections of TA muscle from C57BL/6 and Myf5^Cre/Wt^/Pitx2^−/−^ mice. All the samples were post-fixed in 4% PFA, and blocked for endogenous peroxidase activity by a 10-minute wash done in 0.03% H_2_O_2_/PBS. Subsequently, permeabilization was performed with 2% acetone/PBS for 5 min. After one wash with 30% formamide/2xSSC buffer (300 mM NaCl, 30 mM Na_3_Citarte-2H_2_O, pH 5) for 10 min, pre-hybridization was done for 30 min at 61°C with the hybridization mix (50% formamide, 1,3x SSC, 0.5 mg/ml yeast tRNA, 0.2x CHAPS, 0.5M EDTA NaOH, pH 8, and 0.2%v/v Tween^®^-20). Hybridization was performed with 25 nM of the hsa-miR-106b-5p double DIG-labeled LNA probe (339111 YD00618264-BCG, Qiagen) diluted in hybridization mix at 61°C for 2 h and 30 min. Samples were then washed in 2x SSC and 1x SSC, at 61°C and RT respectively. Then, samples were rinsed with 1x PBS prior to Alexa Fluor™ TyramideSuperBoost™ Kit protocol (B40943 or B40912, Invitrogen), following the manufacturer’s instructions.

### Total RNA Extraction and RT-qPCR Analyses

Muscle total RNA was extracted from treated TA muscles by using Direct-zol™ RNA MiniPrep-Zymo Research kit (Zymo Research, R2050) following manufacturer’s instructions. One microgram of total RNA was reverse transcribed using Maxima First Strand cDNA Synthesis Kit (Thermo Fisher, K1642) following manufacturer’s instructions. As a reverse transcription negative control, each sample was subjected to the same process without reverse transcriptase.

Real-time PCR was performed by using an MxPro Mx3005p PCR thermal cycler (Stratagene, Spain) using SsoFast™ EvaGreen^®^ Supermix (Bio-Rad, 1725201) and primers listed in Table 2. The relative level of expression of each gene was calculated by using the 2-∆∆Ct method (Pfaffl, 2001) with Gapdh and Gusb genes as mRNA normalizers.

**TABLE 2:** qPCR primers.

Gene	Forward primer (5′ to 3′)	Reverse primer (5′ to 3′)
Gapdh (NM_008084.2)	GGC​ATT​GCT​CTC​AAT​GAC​AA	TGT​GAG​GGA​GAT​GCT​CAG​TG
Gusb (NM_010368.1)	ACG​CAT​CAG​AAG​CCG​ATT​AT	ACT​CTC​AGC​GGT​GAC​TGG​TT
Pitx2c (NM_001042502.1)	CCT​CAC​CCT​TCT​GTC​ACC​AT	GCC​CAC​ATC​CTC​ATT​CTT​TC
Pax7 (NM_011039.2)	TCT​TAC​TGC​CCA​CCC​ACC​TA	GTG​GAC​AGG​CTC​ACG​TTT​TT

Each PCR reaction was performed in triplicate and repeated at least in three different biological samples to have a representative average. qPCR program consisted of 95°C for 30 s (initial denaturalization), followed by 40 cycles of 95°C for 5 s (denaturalization); 60°C for 10 s (annealing) and 75°C for 7 s (extension). Finally melt curves were determined by an initial step of 95°C for 5 s followed by 0,5°C increments for 7 s from 65 to 95°C.

### Quantification and Statistical Analysis

For comparison between two groups, two-tailed paired, unpaired Student’s t tests were performed to calculate p-values and to determine statistically significant differences. The number of independent experimental replications (n value ≥3: mice, experiments, wells or counted cells/muscles). Mean ± SD and statistical test (p-value) are reported in each corresponding figure legend. All statistical analyses were performed with GraphPad Prism. Images were processed for quantification with ImageJ software.

## Results

### Pax3^Cre/Wt^/Pitx2^−/−^ Embryos Display Defects on Primary and Secondary Myogenesis and Muscle Hypotrophy

**FIGURE 1:**
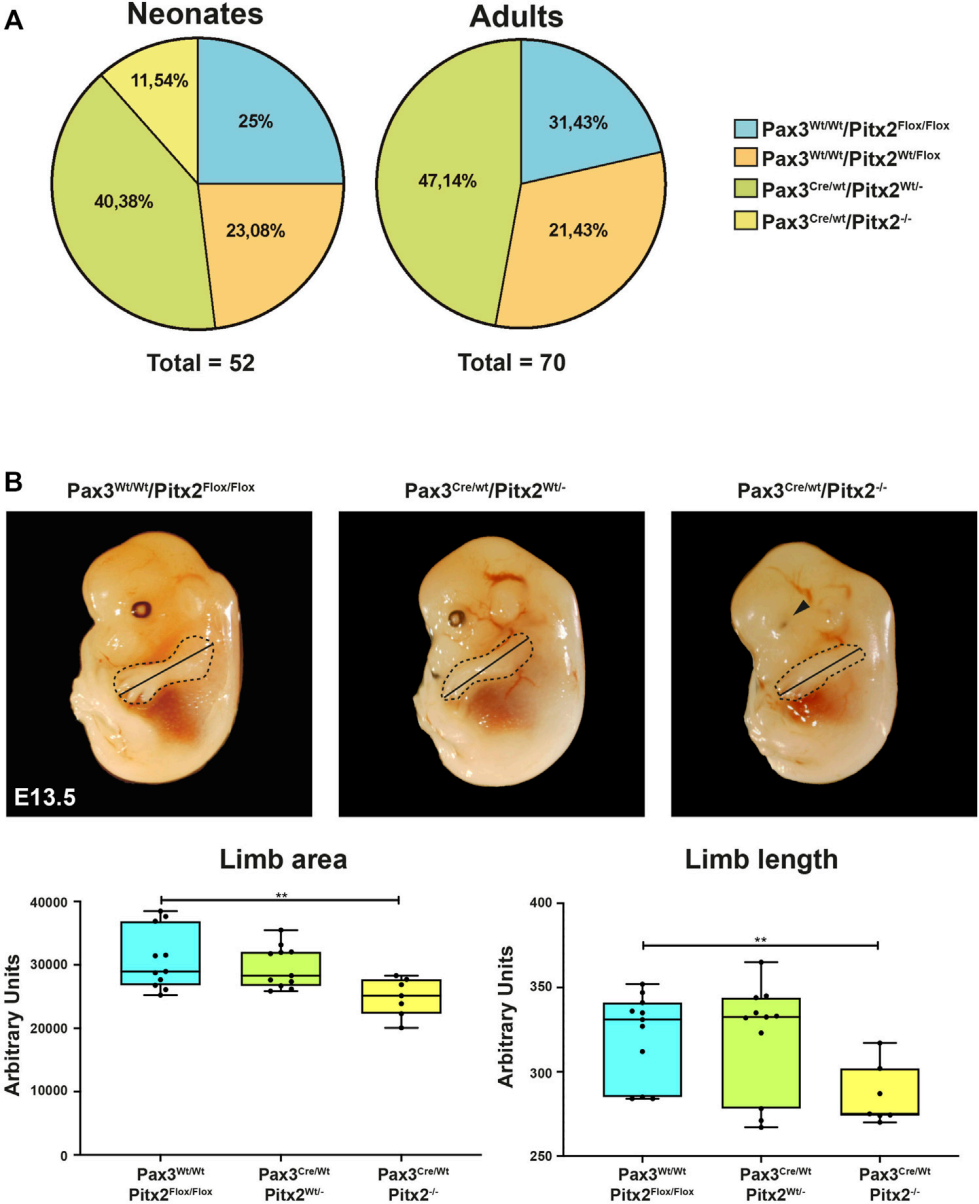
Analysis of Pax3^Cre/Wt^ Pitx2^−/−^ conditional mutants. **(A)** Mendelian ratios and survival rates for Pax3^Wt/Wt^/Pitx2^Flox/Flox^, Pax3^Cre/Wt^/Pitx2^Flox/Wt^, Pax3^Cre/Wt^/Pitx2^Wt/-^ and Pax3^Cre/Wt^ Pitx2^−/−^ mice at neonatal (*n* = 52) and adult stage (*n* = 70). **(B)** Schematic representation of morphometric analyses in Pax3^Cre/Wt^/Pitx2^−/−^ mutant embryos (E13.5) carried out by evaluating limb length (black line) as well as limb areas (delineated by dotted area) by using the image J software (NIHImage) in a total of 40 embryos (*n* = 10 for Pax3^Cre/Wt^/Pitx2^−/−^, *n* = 13 for Pax3^Cre/Wt^/Pitx2^Wt/-^ and *n* = 17 for Pax3^Wt/Wt^/Pitx2^Flox/Flox^). Quantification of limb length, limb area and body are shown.**p < 0.01. Ocular defects are indicated by a black arrow.

Somitic expression of Pitx2 initiates in Pax3-expressing cells in the DMT (Supplementary Figure S1A and (L’honoré et al., 2010) and Pax3 gene is a master regulator for myogenic lineage specification (Buckingham and Rigby, 2014; Chang et al., 2019). To better understand the role of *Pitx2* in early MPCs, we took advantage of a conditional KO approach by crossing mouse strain Pitx2 floxed mice (Pitx2^Flox/Flox^) (Tessari et al., 2008; Chinchilla et al., 2011; Ammirabile et al., 2012) with Pax3^Cre/Wt^ strain allowing specific inactivation of Pitx2 homedomain deletion and inactivation of all Pitx2 isoforms in the early Pax3-expressing myogenic precursor cells. Quantification of Pitx2 mRNA in E10.5 and E12.5 wild type and mutant DMT and limbs respectively indicated a dose-dependent reduction in Pitx2 transcript levels (Supplementary Figure S1B). Inactivation of Pitx2 in Pax3^+^-derived cells lead to perinatal lethality since, although genotype distribution in newborn mice was around to the expected mendelian ratio, no homozygous mice survived at day 1-2 after birth (Figure 1A). We observed that around 40% of homozygous mutant mice on E18.5 showed cardiac defects such as Double outlet right ventricle (DORV) (Supplementary Figure S1C). This cardiac anomaly may be due to the previously reported effects of Pitx2 inactivation in the Pax3^+^ cardiac neural crest cells which contribute to cardiac septation during development (Olaopa et al., 2011). This anomaly could explain, at least in part, the perinatal lethality.

To characterize how the deficiency of Pitx2 in predetermined early myogenic progenitors may affect to embryonic primary myogenesis we have analyzed the limb phenotype as well as muscle progenitor cell distribution in Pax3^Cre/Wt^/Pitx2^−/−^ mutant embryos. Since skeletal muscle progenitor cells originate from somites and DMT between E8.5 and E13.5 in the mouse embryo (Tajbakhsh, 2009), those analyses were carried out at E10.5 and E13.5 developmental stages. Similar to that described for Pitx2 systemic mutant (Lozano-Velasco et al., 2011), no differences in the limb phenotype was observed in mutant embryos at E10.5 (data not shown); however, apparent limb morphological defects in these mutants were detected at E13.5 as observed by a reduction in the limb length and limb area (Figure 1B). In addition, defects on eye development were also observed as previously described for Pitx2 systemic mutants (Gage et al., 1999) (Figure 1B).

**FIGURE 2:**
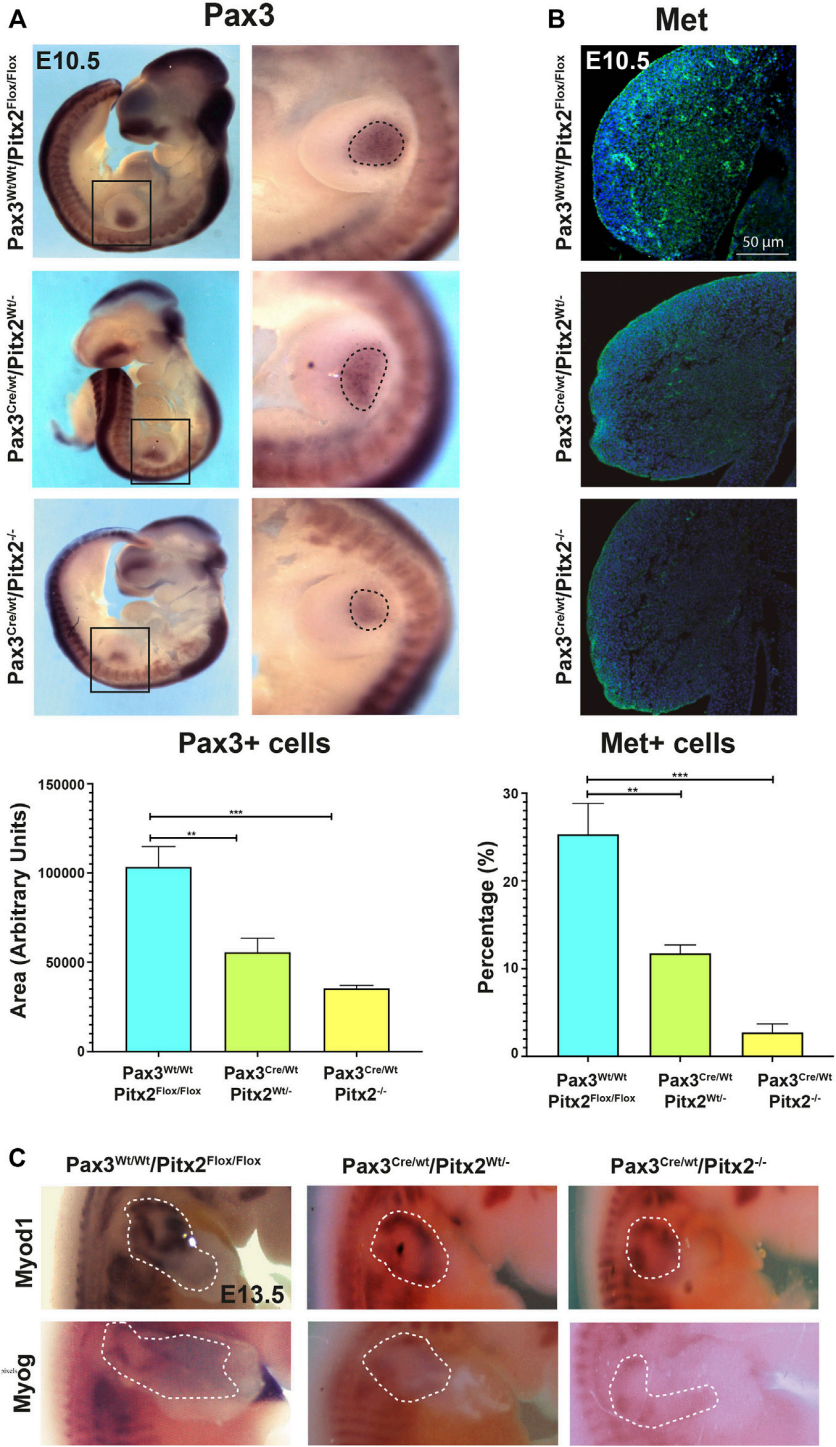
Cell migration of *Pax3* ^+^ precursors is reduced in Pax3^Cre/Wt^ Pitx2^−/−^ conditional mutant embryos. **(A)** Representative images of *in situ* hybridization for *Pax3* in Pax3^Wt/Wt^/Pitx2^Flox/Flox^, Pax3^Cre/Wt^ Pitx2^Wt/-^ and Pax3^Cre/Wt^ Pitx2^−/−^ embryos at E10.5. Pax3^+^ cells in the limb buds are dotted. Quantification of the area of *Pax3*-expressing cells in the limb buds of Pax3^Cre/Wt^ Pitx2^−/−^ conditional mutant embryos is shown. **(B)** Representative images and its magnification of immunohistochemistry for c-MET in Pax3^Wt/Wt^/Pitx2^Flox/Flox^, Pax3^Cre/Wt^ Pitx2^Wt/-^ and Pax3^Cre/Wt^ Pitx2^−/−^embryos at E10.5. Quantification of the number of cMET^+^ cells with respect to total nuclei in the limb buds of Pax3^Cre/Wt^ Pitx2^−/−^ conditional mutant embryos is shown. **(C)** Representative images of *in situ* hybridization for *Myod1* and *Myog* in Pax3Cre^Wt/Wt^/Pitx2^Flox/Flox^, Pax3^Cre/Wt^/Pitx2^Wt/-^ and Pax3^Cre/Wt^/Pitx2^−/−^ at E13.5 stage. *Myod1* and *Myog* expresion zones in the limb buds are dotted. **p < 0.01, ***p < 0.001 (*n* = at least 3 embryos per condition).

We also check for myogenic precursors population by evaluating Pax3 expression; no evident changes in Pax3 expression pattern in somites were detected in Pax3^Cre/Wt^/Pitx2^−/−^ mutant embryos at E10.5 (Figure 2A). However, we observed that the area containing of Pax3^+^ cells in the limb buds was reduced in homozygous conditional mutant embryos with respect to heterozygous and wild type mice at this stage (Figure 2A). To assess if decreased Pax3-expressing cell population in the limb of E10.5 mutant embryos may be due to defects on migration of muscle progenitors, we evaluated migratory MPCs by MET (met proto-oncogene C-Met) staining, a well-recognized marker for migrating Pax3^+^ progenitors (Bladt et al., 1995; Dietrich et al., 1999). We found that the number of migrating MET^+^ cells was significantly decreased in Pax3^Cre/Wt^/Pitx2^−/−^ embryos (Figure 2B) indicating that migration of MPCs is compromised in those mutant mice. In agreement with the idea of a decreased migration of Pax3^+^ myogenic precursors, the expression of the myogenic differentiation markers *Myod1* and *myogenin* (*MyoG*) were lower in the limb buds of Pax3^Cre/Wt^/Pitx2^−/−^ mutant mice at E13.5 (Figure 2C).

**FIGURE 3:**
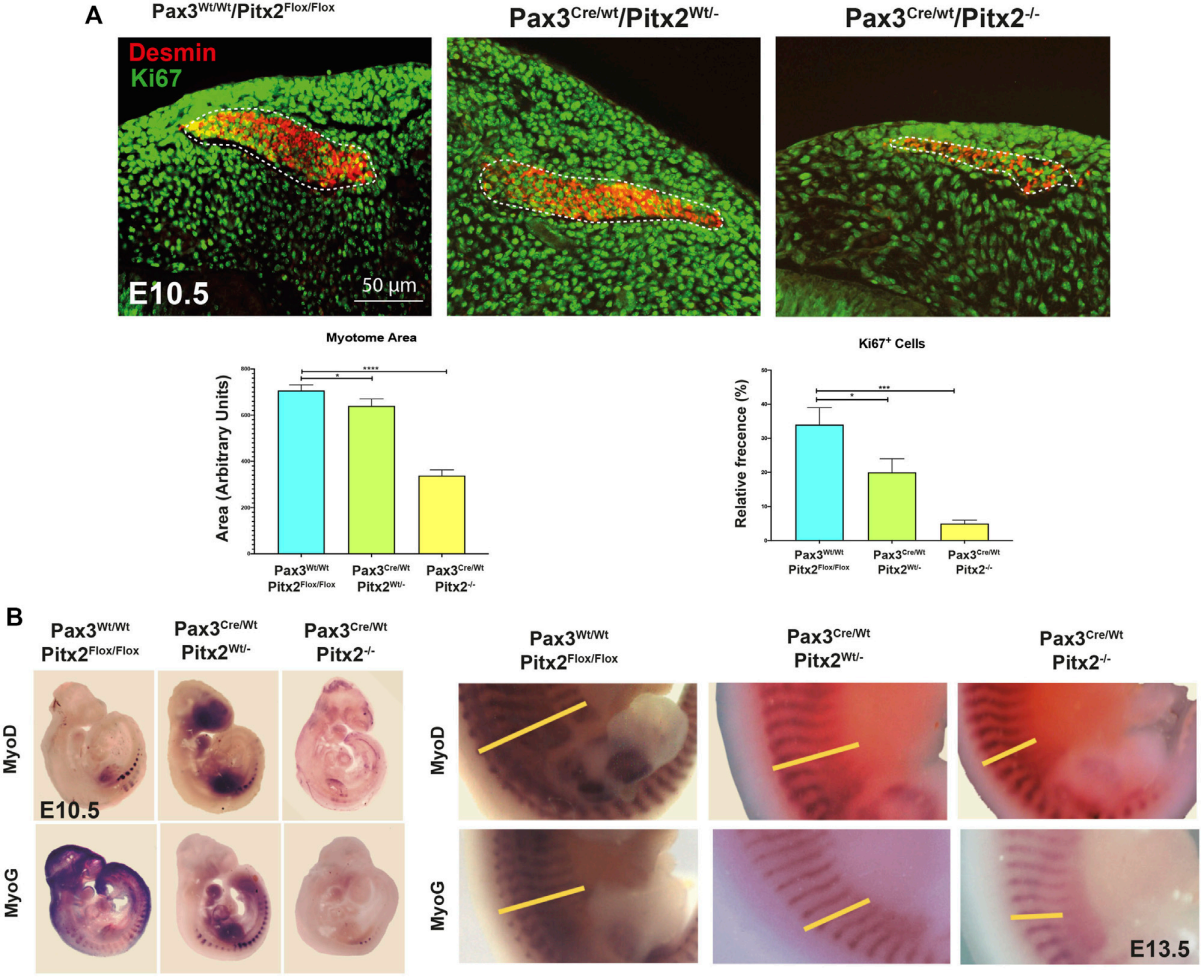
The myotome area as well as the number of Ki67^+^ cells is reduced in Pax3^Cre/Wt^/Pitx2^−/−^conditional mutant embryos. **(A)** Representative images of DESMIN (dotted area) and KI67 co-immunostaining in the myotome of Pax3^Wt/Wt^/Pitx2^Flox/Flox^, Pax3^Cre/Wt^/Pitx2^Wt/-^ and Pax3^Cre/Wt^/Pitx2^−/−^ mice at E10.5 stage. Quantification of Ki67^+^ cells in the myotome is shown. **(B)** Representative images of *in situ* hybridization for *Myod1* and *Myogenin* (*MyoG*) in Pax3^Wt/Wt^/Pitx2^Flox/Flox^, Pax3^Cre/Wt^/Pitx2^Wt/−^and Pax3^Cre/Wt^/Pitx2^−/−^ embryos at E10.5 and E13.5 stages. Note that *Myod1* and *Myogenin* staining *at* E13.5 are delineated by yellow bars. *p < 0.1, ***p < 0.001 (*n* = at least 3 embryos per condition).

In addition, we evaluated MT formation and we observed that Pax3^Cre/Wt^/Pitx2^−/−^ mutant embryos developed smaller MT and, in agreement with our previously reported analysis in Pitx2 systemic mutants (Lozano-Velasco et al., 2011), a clear decrease in the number of proliferative cells (KI67^+^) was detected in the MT of Pax3^Cre/Wt^/Pitx2^−/−^ mutant mice at E10.5 (Figure 3A); reinforcing the notion of a role for Pitx2 controlling cell proliferation in myogenic progenitors. Moreover, consistent with a reduced number of myogenic progenitors in early MT the *Myod1* and *MyoG* staining was reduced in the DMT at E10.5 stages as well as at later stages (E13.5) (Figure 3B). All together those data reveal a requirement of Pitx2 for both cell proliferation and proper migration of MPCs during primary myogenesis.

**FIGURE 4:**
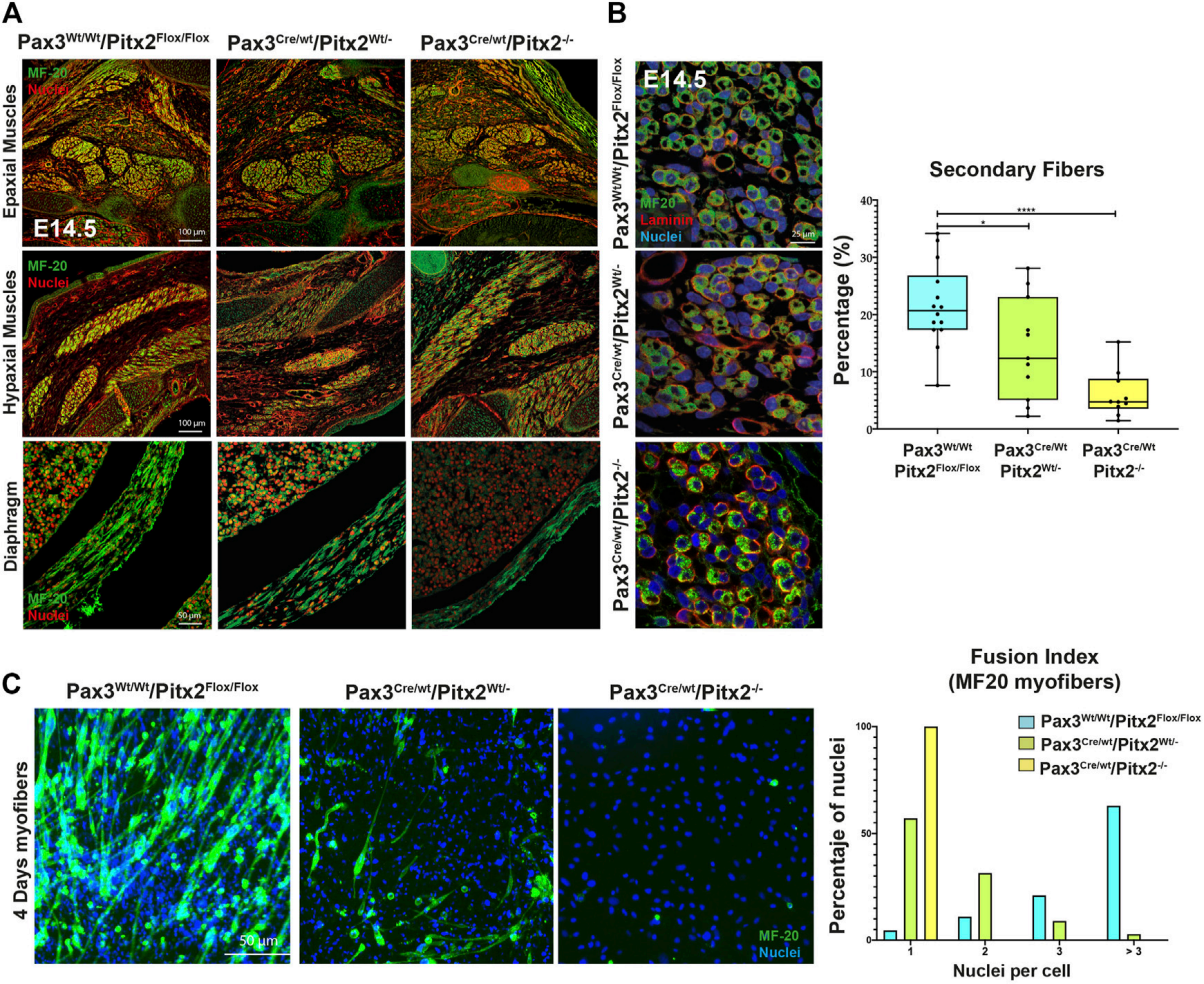
At fetal stages Pax3^Cre/Wt^/Pitx2^−/−^ mutants display muscle hypotrophy together with altered secondary myogenesis and myoblasts differentiation. **(A)** Representative images of MF20 immunostaining in Epaxial, Hypaxial muscles and diaphragm in Pax3^Wt/Wt^/Pitx2^Flox/Flox^, Pax3^Cre/Wt^/Pitx2^Wt/-^ and Pax3^Cre/Wt^/Pitx2^−/−^ embryo at E14.5 stage. **(B)** Representative images of MF20 and Laminin immunostaining in Pax3^Wt/Wt^/Pitx2^Flox/Flox^, Pax3^Cre/Wt^/Pitx2^Wt/-^ and Pax3^Cre/Wt^/Pitx2^−/−^embryo at E14.5 stage. Percentage of secondary myotubes is shown. **(C)** Representative images of cultured myoblasts isolated from the limbs of E18.5 Pax3^Wt/Wt^/Pitx2^Flox/Flox^, Pax3^Cre/Wt^/Pitx2^Wt/-^ and Pax3^Cre/Wt^/Pitx2^−/−^ embryos stained with anti-MHC antibody. Quantification of index fusion is shown. *p < 0.1, ***p < 0.001 (*n* = at least 3 embryos per condition).

To check the consequences of Pitx2 deletion during subsequent fetal myogenesis, we analyzed the epaxial and hypaxial muscle mass at E14.5 by MF20 staining. As observed in Figure 4A, Pax3^Cre/Wt^/Pitx2^−/−^ conditional mutant mice display muscle hypotrophy in epaxial and hypaxial fetal muscle, including the diaphragm (Figure 4A). This observation indicates that neonatal lethality may be also due to severe diaphragm hypotrophy. Beginning at E14.5, secondary myotubes form in tight association with primary myotubes, and they account for much of the muscle growth during fetal development (Buckingham et al., 2003). Thus, in order to evaluate if secondary myogenesis is also affected, the number of MHC^+^ secondary myotubes formed in mutant embryos was analyzed. Our results showed that the number of secondary myotubes (MyHC^+^) were significantly reduced in mutant muscles, but they were frequently observed in wild-type (Figure 4B) indicating that secondary myogenesis is also impaired in these mutant mice. To examine the ability of Pitx2-depleted myoblasts to differentiate and fuse, primary myoblasts were isolated from E18.5 Pax3Cre^Wt/Wt^/Pitx2^Flox/Flox^, Pax3^Cre/Wt^/Pitx2^Wt/-^ and Pax3^Cre/Wt^/Pitx2^−/−^ embryos. Upon selection and expansion of the primary cells in proliferation media, we evaluated the differentiation potential by MF20 staining. We found that myoblasts isolated from Pax3^Cre/Wt^/Pitx2^−/−^ embryos display a lower capability to generate MF-20 positive myofibers (Figure 4C) and were unable to form long multinucleated fibers after 4 days in differentiating conditions in contrast to Pax3^Wt/Wt^/Pitx2^Flox/Flox^ myoblasts. Quantification of the fusion index revealed that all of MF20- positive myoblasts isolated from Pax3^Cre/Wt^/Pitx2^−/−^ conditional mutant mice remained mononucleated. A portion of the cells isolated from Pax3^Cre/Wt^/Pitx2^Wt/-^ conditional mutant embryos (31,42%) could form binucleated and (8,57%) trinucleated syncytia after differentiation (Figure 4C). These results showed that inactivation of Pitx2 finally also interferes with myoblast differentiation.

**FIGURE 5:**
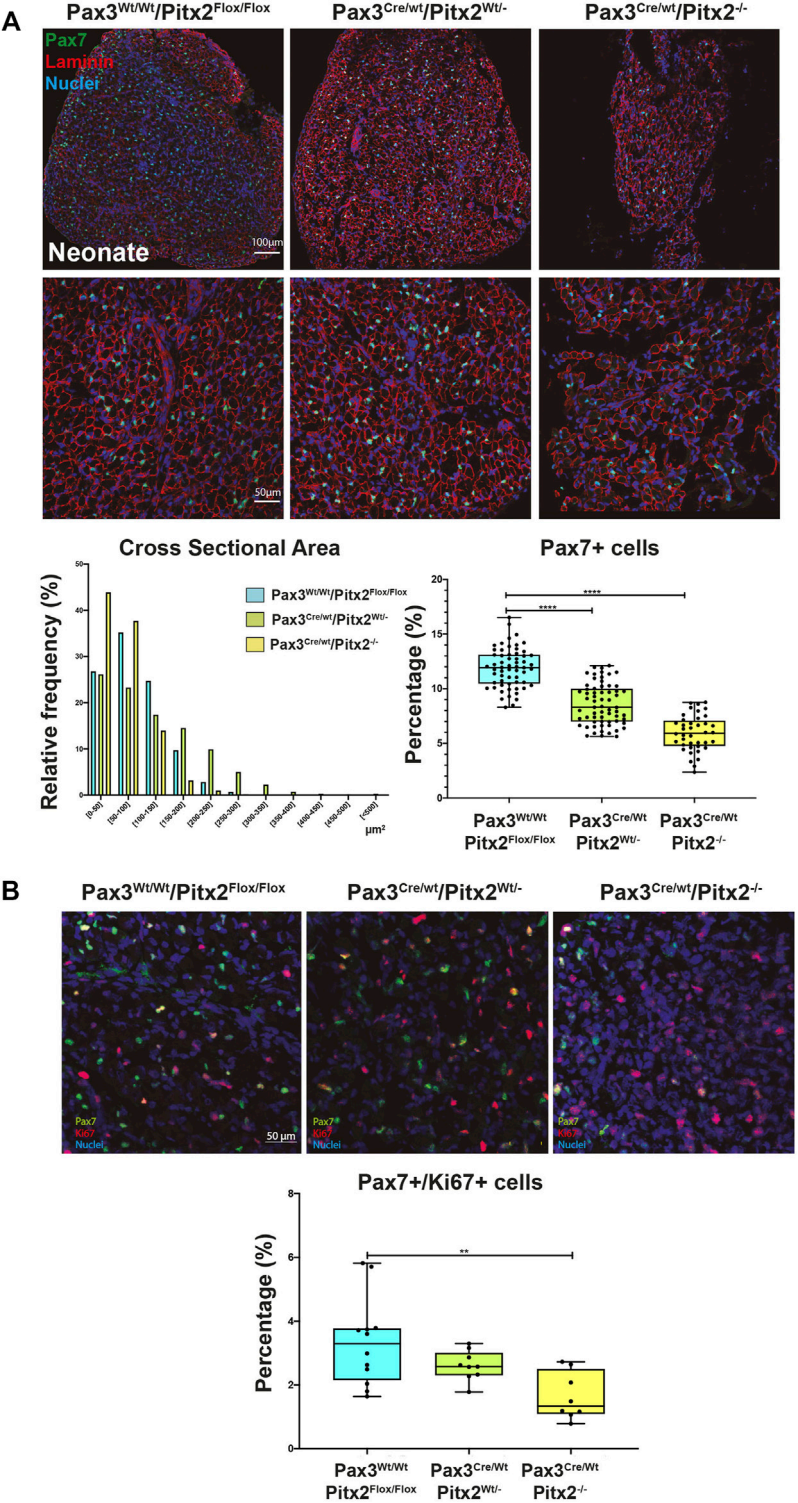
Neonates of Pax3^Cre/Wt^/Pitx2^−/−^ conditional mutant show reduced muscle size and a lower amount of proliferative SC. **(A)** Representative images of PAX7 and Laminin co-immunostaining in the TA muscles of Pax3^Wt/Wt^/Pitx2^Flox/Flox^, Pax3^Cre/Wt^/Pitx2^Wt/-^ and Pax3^Cre/Wt^/Pitx2^−/−^ mice at neonatal stage. Distribution of cross-sectional area in tibialis anterior (TA) muscles and percentage of PAX7^+^ cells are shown. **(B)** Representative images of PAX7 and KI67 co-immunostaining in the TA muscles of Pax3^Wt/Wt^/Pitx2^Flox/Flox^, Pax3^Cre/Wt^/Pitx2^Wt/-^ and Pax3^Cre/Wt^/Pitx2^−/−^ mice at neonatal stage. Percentage of PAX7^+^/KI67^+^ cells is shown. **p < 0.01, ****p < 0.0001(*n* = at least 3 neonates per condition).

Muscle hypotrophy persists in this Pax3^Cre/Wt^/Pitx2^−/−^ conditional mutant at neonatal stages as observed by a decrease muscle size together with a shift in the distribution of the fiber size to the lowest area classes (Figure 5A). Since PAX3^+^/PAX7^+^ MPCs are the major source of adult SCs in trunk and limb muscles, we also looked at whether SC population was also affected in neonatal Pax3^Cre/Wt^/Pitx2^−/−^ conditional mutant and, as illustrated in Figure 5A, the percentage of SCs within the neonatal muscle was also decreased. This finding might be a consequence of decreased migration of MPCs observed in those conditional mutant mice at early stages and indicate that Pitx2 inactivation in Pax3-expressing precursors also impacts the pool of SC population that remain in the adult muscle. Besides, after birth, an extensive skeletal muscle growth occurs supported by the proliferation of PAX7^+^ cells (White et al., 2010; Bachman et al., 2018). Therefore, to further elucidate if the lack of Pitx2 also target cell proliferation of PAX7^+^ neonatal SCs, we analyzed the number of proliferative cells by co-immunostaining with Ki67. As illustrated in Figure 5B, the number of PAX7^+^/KI67^+^ cells were clearly lower in Pax3^Cre/Wt^/Pitx2^−/−^ mice than in Pax3^Wt/Wt^/Pitx2^Flox/Flox^ and Pax3^Cre/Wt^/Pitx2^Wt/-^ mice, revealing that neonatal or juvenile SCs of Pax3^Cre/Wt^/Pitx2^−/−^ conditional mutant mice also display cell proliferation defects.

### Regeneration-Related Activity Is Diminished in Pax3^Cre/Wt^/Pitx2^Wt/-^ Heterozygous Mice

**FIGURE 6:**
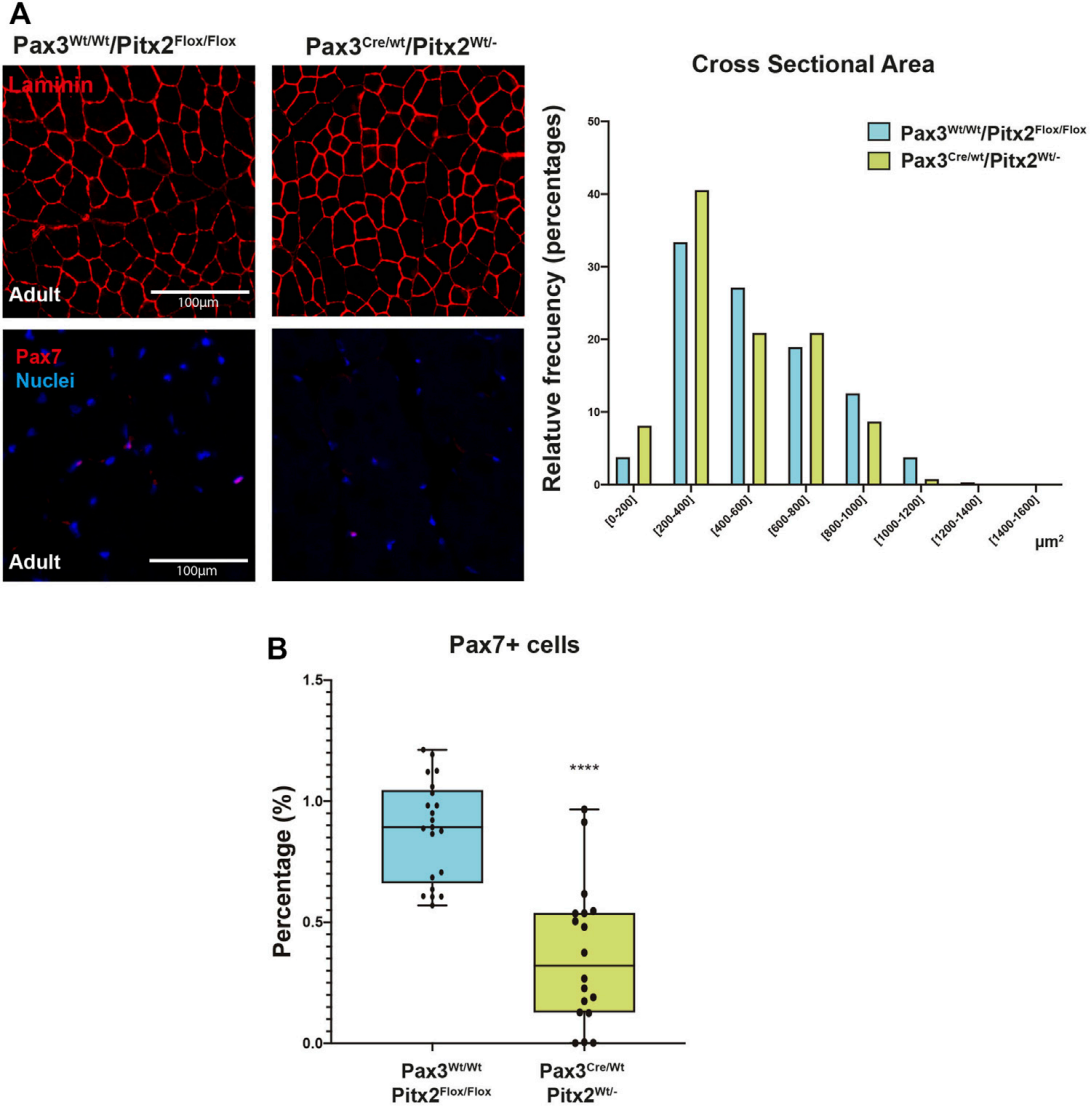
Pax3^Cre/Wt^/Pitx2^Wt/-^ conditional mutant adults show reduced CSA as well as minor population of SC. **(A)** Representative images of PAX7 and LAMININ in the TA muscles of Pax3^Wt/Wt^/Pitx2^Flox/Flox^, Pax3^Cre/Wt^/Pitx2^Wt/Wt^ and Pax3^Cre/Wt^/Pitx2^Wt/-^ mice at adult stage **(A,B)**. Distribution of cross-sectional area in tibialis anterior (TA) muscles. Percentage of PAX7^+^ cells is shown. **p < 0.01, ****p < 0.0001 (*n* = at least 3 mice per condition).

Since we have previously reported that Pitx2-overexpression can modify the myogenic potential of adult SCs (Vallejo et al., 2018), we decide to further evaluate if *Pitx2* deficit in early myogenic precursors also impacts on SC behavior. For this analysis we have used adult Pax3^Cre/Wt^/Pitx2^Wt/-^ heterozygous mutant mice whose *Pitx2* expression is reduced by around 50% (Supplementary Figure S2A). As observed in Figure 6A, the muscles of heterozygous adult mutant mice maintained a shift in the distribution of the fiber size to the lowest area classes as well as a reduced number of PAX7^+^ SCs (Figure 6B).

**FIGURE 7:**
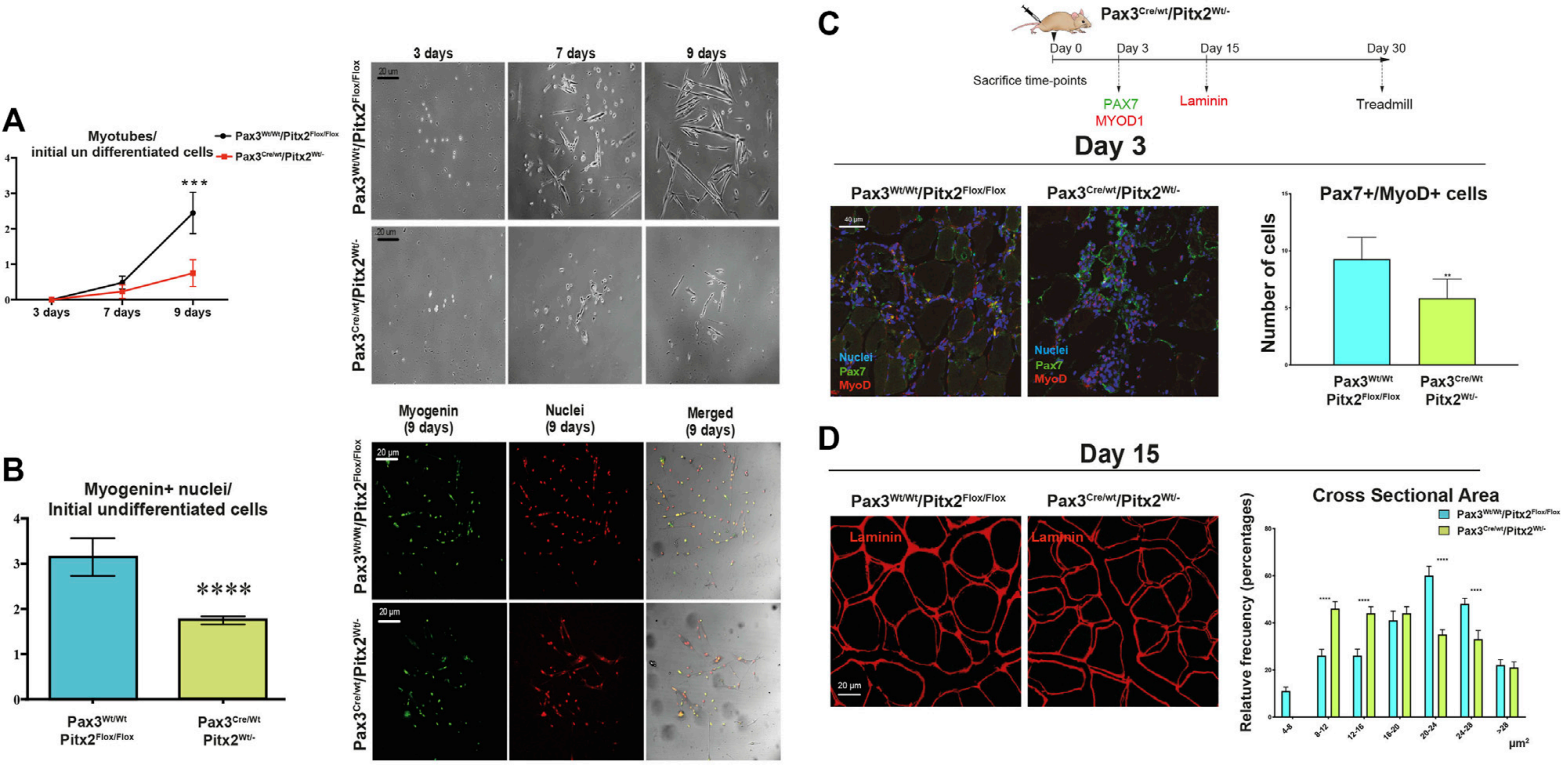
SC differentiation and muscle regeneration are compromised in adult Pax3^Cre/Wt^/Pitx2^Wt/-^ heterozygous mice. **(A)** Ratio of number of myotubes per initial undifferentiated cells (day 3 of culture) through *in vitro* differentiation (7 and 9 days of culture) in SC isolated from Pax3^Wt/Wt^/Pitx2^Flox/Flox^ vs*.* SC from Pax3^Cre/Wt^/Pitx2^Flox/,^ and representative images of *in vitro* differentiation. **(B)** Relative values of number of myogenin + nuclei in differentiated cells (day 9 of culture) per initial undifferentiated cells in SC isolated from Pax3^Wt/Wt^/Pitx2^Flox/Flox^ vs*.* SC from Pax3^Cre/Wt^/Pitx2^Flox/,^ and representative images of myogenin + nuclei. **(C)** Scheme of CTX injection in the TA of Pax3^Cre/Wt^/Pitx2^Wt/Wt^ and Pax3^Cre/Wt^/Pitx2^Wt/-^ mice and representative image of PAX7^+^/MYOD1^+^ cells at day 3 after cardiotoxin injection in tibialis anterior muscle (TA). Percentage of PAX7^+^/MYOD1^+^ cells is shown. **(D)** Representative images of LAMININ staining in tibialis anterior (TA) muscles of Pax3^Cre/Wt^/Pitx2^Wt/-^ heterozygous mice vs. Controls Pax3^Cre/Wt^/Pitx2^Wt/-^ mice at day 15 after cardiotoxin injection. Cross-sectional area is shown.*p < 0.0.1, ***p < 0.001, ****p < 0.0001 (*n* = at least 3 mice per condition).

Importantly, we observed that cultured of SC isolated from Pax3^Cre/Wt^/Pitx2^Wt/-^ mice displayed a reduced capability to form differentiated myotubes *in vitro* (Figure 7A) and a decreased number of MYOGENIN^+^ cells (Figure 7B) indicating that the loss of *Pitx2* function in MPCs diminished their capability to form myotubes and reinforce the notion that Pitx2 modulates SC differentiation.

To further investigate whether *Pitx2* inactivation may affect to SC function during muscle regeneration *in vivo*, we induced skeletal-muscle damage by cardiotoxin injection (CTX) in the tibialis anterioris (TA) of 4-month-old Pax3^Cre/Wt^/Pitx2^Wt/-^ heterozygous mice and Pax3^Wt/Wt^/Pitx2^Flox/Flox^ control mice. After skeletal-muscle injury, we evaluated the impact of genetic loss of Pitx2 on the myogenic response during the initial waves of muscle regeneration by analyzing the number of PAX7^+^/MYOD1^+^ SCs at 3 days after damage. As illustrated in Figure 7C the number of activated (PAX7^+^/MYOD1^+^) SCs was lowered in injured mutant muscles. Moreover, regeneration was impaired, as indicated by smaller myofiber size 15 days after muscle injury (Figure 7D), emphasizing the requirement of *Pitx2* for *in vivo* myogenic differentiation.

### The Muscles of Myf5^Cre/Wt^/Pitx2^−/−^ Mutant Mice Display Differences in the Amount of Myf5^+^ and miR106b^+^ SC Subpopulations Compromising Differentiation

**FIGURE 8:**
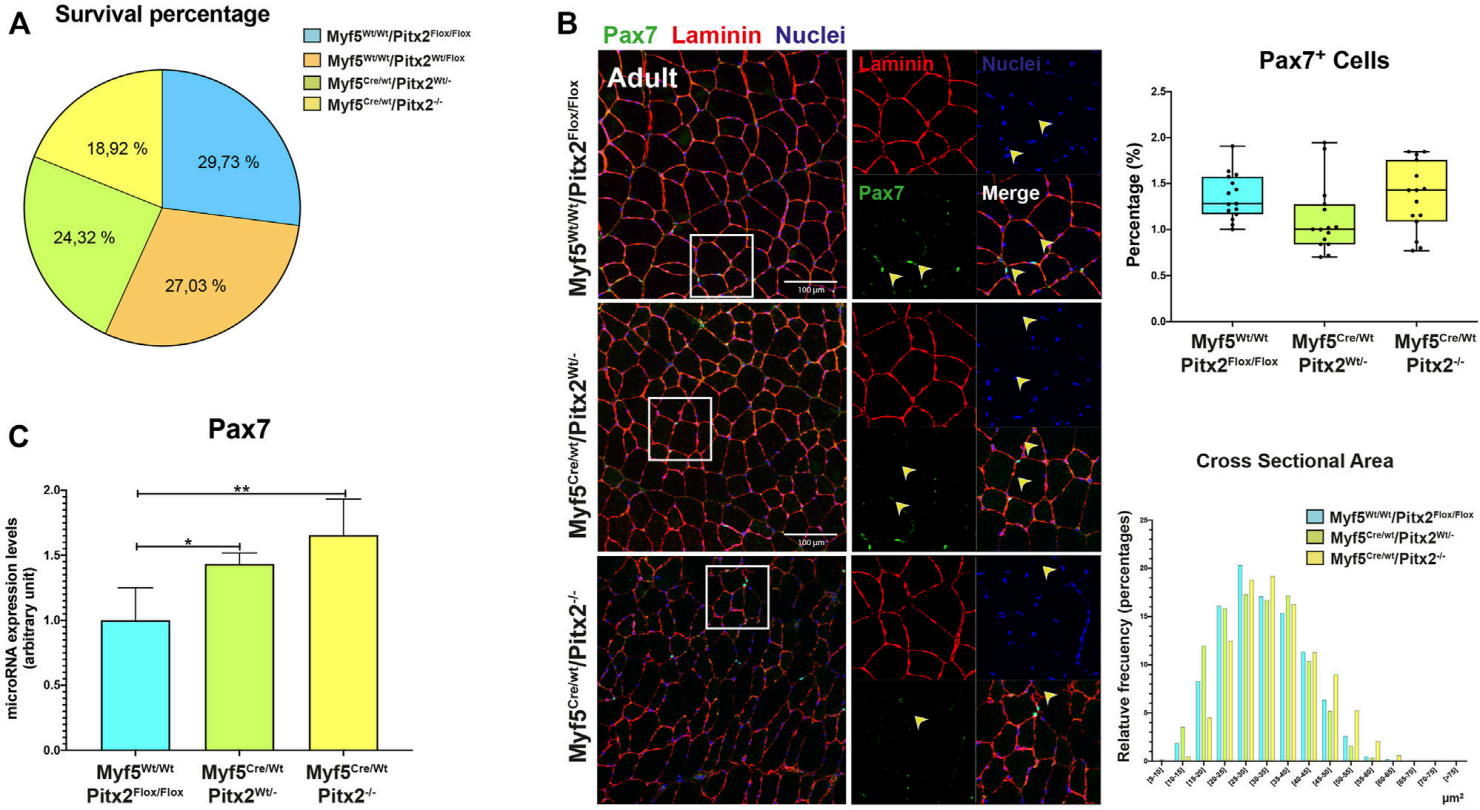
Myf5^Cre/Wt^/Pitx2^−/−^ mice have normal survival rates but possess satellite cells with high *Pax7* expression. **(A)** Survival rates for Myf5^Wt/Wt^/Pitx2^Flox/Flox^, Myf5^Wt/Wt^/Pitx2^Flox/Wt^, Myf5^Cre/Wt^/Pitx2^Wt/-^ and Myf5^Cre/Wt^/Pitx2^−/−^ mice. **(B)** Representative images of PAX7 and LAMININ co-immunostaining in the TA muscles of Myf5^Wt/Wt^/Pitx2^Flox/Flox^, Myf5^Cre/Wt^/Pitx2^Wt/-^ and Myf5^Cre/Wt^/Pitx2^−/−^ mice and quantification of PAX7^+^ cells. **(C)** qRT-PCR analyses for *Pax7* in the TA muscles of Myf5^Wt/Wt^Pitx2^Flox/Flox^, Myf5^Cre/Wt^/Pitx2^Wt/-^ and Myf5^Cre/Wt^/Pitx2^−/−^ mice. *p < 0.1, **p < 0.01 (*n* = at least 3 mice per condition).

The first sign of myogenic compromise during development is the activation of the myogenic factor *Myf5* in the progenitor cells into somites; and it has been previously established that adult SCs derive from progenitors that express the myogenic determination gene *Myf5* during fetal stages of myogenesis (Relaix et al., 2003; Biressi et al., 2013). Since Pitx2 is also expressed in Myf5+ muscle precursors (Supplementary Figure S1A and L’honoré et al., 2010), to full address the role of *Pitx2* in the committed muscle precursors and in SCs progenitors during skeletal myogenesis, we genetically ablated *Pitx2* in myogenic committed progenitor cells by intercrossing a *Pitx2* floxed mouse line with a *Cre* deleter mouse line (B195AP-Cre)**,** which rendered muscle-lineage-specific *Myf5Cre* recombination (Naldaiz-Gastesi et al., 2016). Mice lacking Pitx2 activity by using a *Myf5Cre* deleter mouse line developed into normal adults and their muscles to have normal fiber size (Figures 8A,B). qRT-PCR analyses of Pitx2 expression in the limb-muscles of mutant embryos revealed that Pitx2 transcript levels were reduced approximately 90% (Supplementary Figure S1B).

**FIGURE 9:**
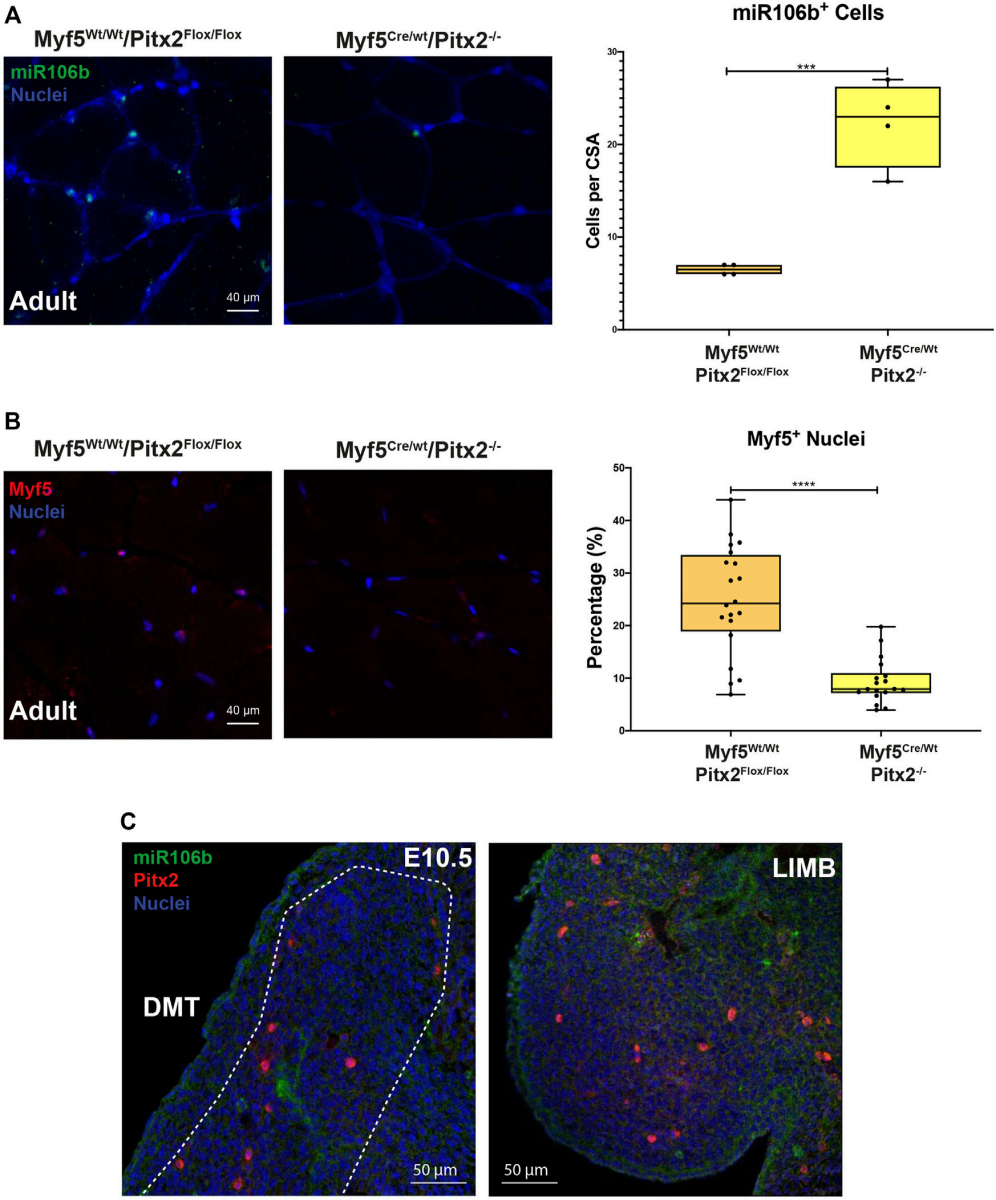
The muscles of Myf5^Cre/Wt^ Pitx2^−/−^ mice have high amounts of miR-106b+ cells but lower numbers of Myf5 cells. **(A)** Representative images of miR-106b *in situ* hybridization (FISH) in the TA muscles of Myf5^Cre/Wt^/Pitx2^Wt/Wt^ and Myf5^Cre/Wt^/Pitx2^−/−^ mice and quantification of miR-106b+ cells. **(B)** Representative images of MYF5^+^ cells in the TA muscles of Myf5^Cre/Wt^/Pitx2^−/−^ conditional mutants and quantification of MYF5+ cells. **(C)** Representative images of FISH for miR-106b and Pitx2 immunostaining in c57 wild-type embryos at E10.5. ***p < 0.001, ****p < 0.0001 (*n* = at least 3 mice per condition).

To check if the lack of Pitx2 function in *Myf5* progenitor cells may affect the SC population in the adult muscle; we first performed PAX7 and LAMININ co-immunostaining in 4 months old Myf5^Cre/Wt^/Pitx2^−/−^ conditional mutants. Quantification of the number of PAX7^+^ sublaminar cells showed no differences in the total number SCs in the muscle Myf5^Cre/Wt^/Pitx2^−/−^ homozygous mutant mice respect to heterozygous and wild-type mice (Figure 8B). However, qRT-PCR analyses for Pax7 expression levels revealed that the level of *Pax7* expression was two-fold increase in Myf5^Cre/Wt^/Pitx2^−/−^ homozygous mutant mice respect to wild-type (Figure 8C) indicating that SCs in these mutants exhibit high levels of *Pax7* expression. High *Pax7* expression is linked to quiescence in SCs (Olguin and Olwin, 2004; Zammit et al., 2006) whereas SC expressing Myf5 are more prone to myogenic differentiation (Kuang et al., 2007); and we have previously described a role of *Pitx2* regulates SC activation and myogenic commitment by repressing miR-106b, a miRNAs that suppress proliferation and myogenic commitment in SCs by targeting cell cycle regulatory genes *Cyclin D1*, *Cyclin D2* and *Myf5* (Lozano-Velasco et al., 2015). Thus, we looked if the lack of Pitx2 in those mutants affects the number of SCs expressing MYF5 and/or miR-106b SCs. As expected, Pitx2 deficiency in Myf5^+^ progenitors lead to an increase in the number of miR-106b^+^ cells in the muscle (Figure 9A). By contrast, the number of MYF5^+^ SCs was clearly diminished in Myf5^Cre/Wt^/Pitx2^−/−^ homozygous mutant mice (Figure 9B). These results suggest that the interplay between Pitx2 and miR-106b expression in myogenic progenitors may be defining different subpopulations of SC precursors during skeletal myogenesis. In agreement with that idea, two clearly distinguishable PITX2^+^ and miR-106b^+^ cell populations may be observed in the DMT and limbs of the mouse embryo at E10.5 (Figure 9C).

### Myf5^Cre/Wt^/Pitx2^−/−^ Mutant Mice Exhibit Severe Defects on Muscle Regeneration

**FIGURE 10:**
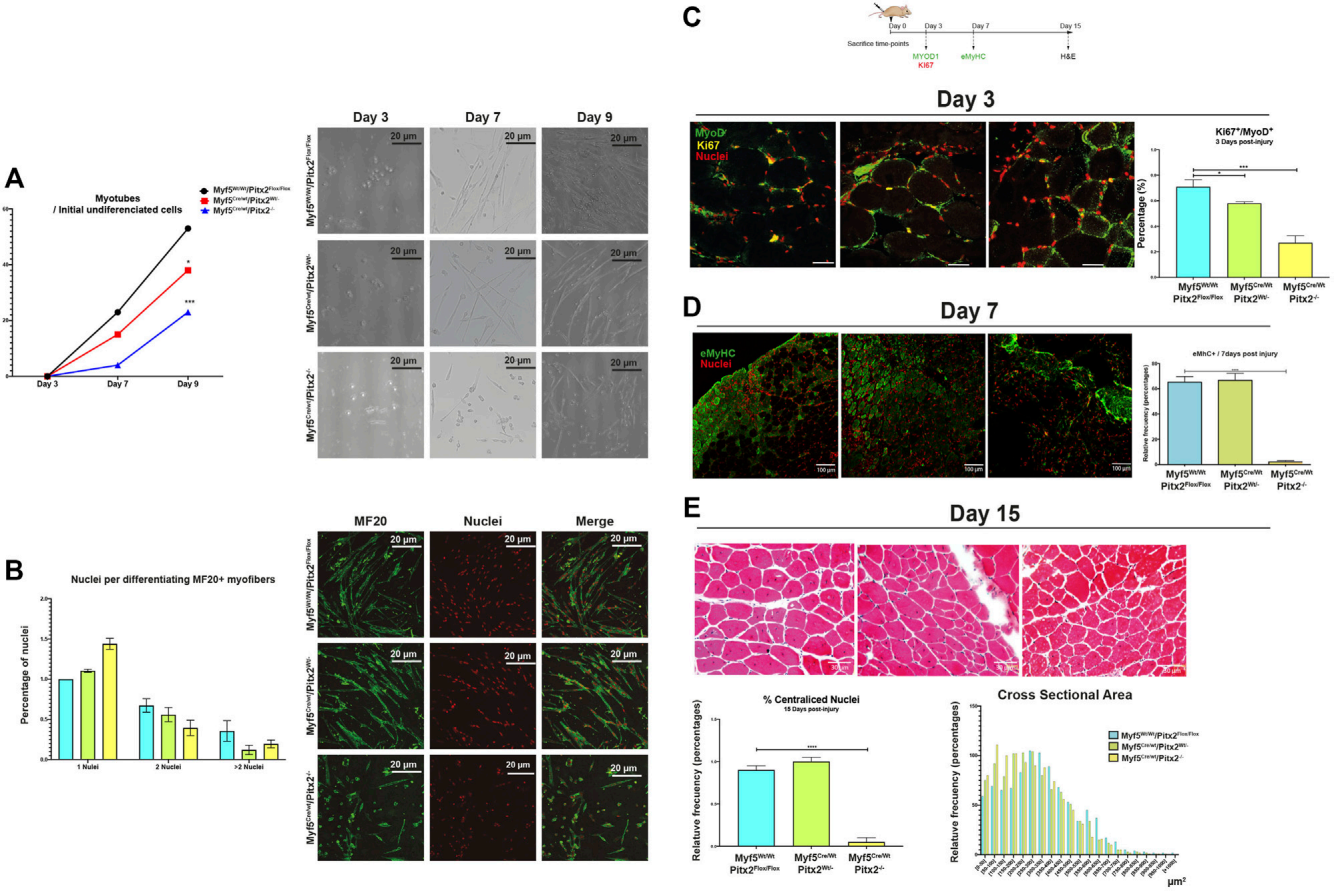
Myf5^Cre/Wt^/Pitx2^−/−^ mice show altered muscle regeneration. **(A)** Ratio of number of myotubes per initial undifferentiated cells (day 3 of culture) through *in vitro* differentiation (7 and 9 days of culture) in SC isolated from Myf5^Wt/Wt^/Pitx2^Flox/Flox^, Myf5^Cre/Wt^/Pitx2^Wt/-^ and Myf5^Cre/Wt^/Pitx2^−/−^ mice. Representative images of *in vitro* differentiation. **(B)** Representative images of MF20 immunostaining in 3 days cultured myoblasts from Myf5^Wt/Wt^/Pitx2^Flox/Flox^, Myf5^Cre/Wt^/Pitx2^Wt/-^ and Myf5^Cre/Wt^/Pitx2^−/−^ mice. Percentage of nuclei per MF20+ myofiber is shown. **(C)** Scheme of CTX injection in the TA of C57BL/6 mice Myf5^Wt/Wt^/Pitx2^Flox/Flox^, Myf5^Cre/Wt^/Pitx2^Wt/-^ and Myf5^Cre/Wt^/Pitx2^−/−^ mice and representative image of KI67^+^/MYOD1^+^ cells at day 3 after cardiotoxin injection. Percentage of KI67^+^/MYOD1^+^ cells is shown. **(D)** Representative image of eMyHC staining at day 7 after cardiotoxin injection in tibialis anterior muscle (TA) of Myf5^Wt/Wt^/Pitx2^Flox/Flox^, Myf5^Cre/Wt^/Pitx2^Wt/-^ and Myf5^Cre/Wt^/Pitx2^−/−^. Percentage of eMyHC^+^ myofibers is shown. **(E)** Representative image of Hematoxilin-Eosin staining at day 15 after cardiotoxin injection in tibialis anterior muscle (TA) of Myf5^Wt/Wt^/Pitx2^Flox/Flox^, Myf5^Cre/Wt^/Pitx2^Wt/-^ and Myf5^Cre/Wt^/Pitx2^−/−^. Percentage of centralized nuclei as well as Cross Sectional Area Distribution at day 15 after cardiotoxin injection are shown^-^. *p < 0.1, ***p < 0.001, ****p < 0.001 (*n* = at least 3 mice per condition).

On account of the maintenance of high levels of *Pax7* expression and the presence of miR-106b on SCs might alter proper myogenic terminal differentiation finally affecting its regenerative capacity (Olguin and Olwin, 2004; Olguin et al., 2007; von Maltzahn et al., 2013; Lozano-Velasco et al., 2015); we next explored SC differentiation capability *in vitro* and regenerative potential *in vivo* in Myf5^Cre/Wt^/Pitx2^−/−^ mutant mice. Hence, we observed that the capability to form myotubes was clearly diminished in SCs isolated from Myf5^Cre/Wt^/Pitx2^−/−^ mutant mice (Figure 10A) and a decreased amount of differentiating MF20^+^ myofibers as well as a lower number of nuclei per MF20^+^ myofiber was also observed (Figure 10B).

In addition, SC regenerative potential was checked by cardiotoxin-induced skeletal-muscle damage in 4-month-old Myf5^Cre/Wt^/Pitx2^−/−^ homozygous, Myf5^Cre/Wt^/Pitx2^Wt/-^ heterozygotes and Myf5^Wt/Wt^/Pitx2^Flox/Flox^ control mice. We found that SCs of Myf5^Cre/Wt^/Pitx2^−/−^ mice display a lower propensity to proliferate at day 3 of muscle injury, as observed by the lowest number of MYOD1^+^/KI67^+^ cells (Figure 10C), which suggests that the inactivation of Pitx2 by using Myf5Cre deleter decrease the functional ability of SCs to activate and participate in tissue repair.

We also tested tissue regeneration by analyzing the newly formed myofibers by using an eMyHC antibody on day 7 of cardiotoxin injection. As observed in Figure 10D, the number of eMyHC^+^ myofibers was dramatically decreased in the injured muscles of Myf5^Cre/Wt^/Pitx2^−/−^ homozygous respect to Myf5^Cre/Wt^/Pitx2^Wt/-^ heterozygotes and Myf5^Wt/Wt^ Pitx2^Flox/Flox^ control mice. The histological analysis of the TA at 15 days after muscle damage clearly showed a lower percentage of fibers with centralized nuclei (Figure 10E) as well as a shift in the distribution of the regenerating fiber size to the lowest area classes (Figure10E). Together, these findings indicate that the lack of Pitx2 un Myf5^+^ myogenic progenitors generates SCs with diminished differentiation capability, thus altering their regenerative potential.

## Discussion

Several previous evidences have revealed that the transcription factor *Pitx2* is a key element involved in the fine-tuning mechanism that regulates skeletal-muscle development as well as the differentiation and cell fate of SCs in the adult muscle (Kioussi et al., 2002; Baek et al., 2003; Martínez-Fernandez et al., 2006; Abu-Elmagd et al., 2010; L’honoré et al., 2010). The phenotypic analyses of Pitx2 systemic mutants as well as genetic ablations by using different skeletal muscle drivers have shown that Pitx2, together with Pitx3, plays an early role prior to terminal differentiation (L’honoré et al., 2014b; L’honoré et al., 2014a). However, the role of *Pitx2* in the progression from early myogenic progenitors to myoblasts, including SCs precursors, remains unsolved. In this study we have generated two conditional *Pitx2* mutant mice to differentially inactivate Pitx2 at the onset of myogenic specification (Pax3^Cre/Wt^/Pitx2^−/−^ mutant mice) and/or after the acquisition of myogenic fate (Myf5^Cre/Wt^/Pitx2^−/−^ mutant mice). Our analyses revealed that Pax3^Cre/Wt^/Pitx2^−/−^ mutants displayed impaired myogenesis; however, in Myf5^Cre/Wt^/Pitx2^−/−^ mutant mice the muscles were normally formed indicating that Pitx2 is required for proper myogenesis in the early uncommitted myogenic precursors but dispensable once myogenic commitment have taken place.

Several seminal works have suggested that Pitx2 could be acting downstream of Pax3 and in parallel with Myf5, at least in the myotome (L’Honoré et al., 2007; Lagha et al., 2010; L’honoré et al., 2010). In this context, L’Honore et al. (2010) have previously showed that systemic Pitx2^−/−^ null mutants display a delay in the onset of Myod1 and Myog expression in the limb buds but not in the myotome, thus they proposed that Pitx2 pathway is of primary importance for limb myogenesis but the Myf5 and Mrf4 pathway predominating in myotome (L’honoré et al., 2010). However, here we show that loss of Pitx2 in early myogenic progenitors lead to a decreased cell proliferation in the early myotome (E10.5). Those data are in agreement with our previous reported findings in myoblasts and Pitx2c^−/−^ systemic mutant indicating a role of Pitx2 in cell proliferation (Lozano-Velasco et al., 2011; Lozano-Velasco et al., 2015) and revealed that Pitx2 also control cell division in the early myotome, further reinforcing the notion of Pitx2 acts modulating cell division in myogenic progenitor cells.

Importantly, we also observed that the amount of Pax3^+^ progenitors that reach the limb buds is decreased in Pax3^Cre/Wt^/Pitx2^−/−^ conditional mutant embryos at early embryonic stages; besides, we also observed that the number of migrating progenitors (Met^+^ cells) was clearly lower in those mutant mice. Together, these data support the idea of a requirement of the Pitx2 function for proper migration of Pax3^+^ myogenic precursor cells. Previous *in vitro* analysis by using myoblasts isolated from limbs of Lbx^EGFP^ mouse indicated that *Pitx2* might influence myoblast cell movements by influencing their polarity and shape (Campbell et al., 2012). Our results provide additional evidence for a Pitx2 requirement for proper migration of uncommitted muscle precursors. Additional studies, by analyzing cell motility mechanisms, will help us to better understand how *Pitx2* operates controlling migration of early myogenic cells. With further development (E12.5) we have detected a lower Myod1^+^ and Myog^+^ staining in the dermomyotome and in the limb buds of Pax3^Cre/Wt^/Pitx2^−/−^ conditional mutant embryos and a clear muscle hypotrophy and foetal stages. We think that those findings might be a consequence of a previous reduced cell proliferation and migration of myogenic progenitors that, ultimately, lead to a decreased amount of differentiating myoblasts. In addition, our *in vitro* analyses showed that mutant foetal myoblast display defects in cell differentiation and fusion. This observation is in accordance with previous analyses that identify Pitx2 target genes that function as components of the assembly, organization and regulation of the cytoskeleton in myogenic cells isolated from limb buds (Campbell et al., 2012) further supporting the notion of a *Pitx2* function for myogenic differentiation.

Muscle hypotrophy as well as a reduced number of SC is also present in the adult muscle of Pax3^Cre/Wt^/Pitx2^Wt/-^ heterozygous mice. Experiments of induced muscle injury in these heterozygous mice demonstrated that their muscular regenerative capacity was highly compromised. Those results are in agreement with our previous reported work highlighting the relevance of Pitx2 in the context of muscle regeneration (Vallejo et al., 2018). Overall our results indicate that the deficiency of Pitx2 in multipotent (Pax3^+^) myogenic progenitors finally determine the number of SC that reach their niche in the adult muscle and their response under injury, supporting the notion that Pitx2 somehow might play a role modulating SC precursors during development. It has been proposed that SCs can originate from different cell progenitor source: Somitic “stem” cell (Pax3^+^/Pax7^+^) and/or foetal myoblast (Pax7^+^/Myf5^+^/Myod1^+^) (Zammit, 2008). Thus, the fact that the lack of Pitx2 in Pax3^+^ MPCs affects both SC number and function in the adult highlight the concept that proper early molecular signals in Somitic “stem” cell (Pax3^+^/Pax7^+^) are required for the acquisition of an adequate and functional SC population in the adult muscle.

Contrary to Pax3^Cre/Wt^/Pitx2^−/−^ conditional mutant; Myf5^Cre/Wt^/Pitx2^−/−^ conditional mutant mice developed into normal adults and their muscles appear to have normal fiber size, indicating that the lack of Pitx2 function in myogenic committed progenitors have not consequences in the developing muscle. It has been previously shown in Pitx2 systemic mutant mice that Myf5 compensates functionally for Myod1 expression in Pitx2^–/–^ limbs (L’honoré et al., 2010). Therefore, consistent with that idea, the lack of Pitx2 in Myf5 expressing myogenic progenitors has not severe consequences in muscle development reinforcing the notion that Pitx2 may be acting in a parallel pathway to Myf5 in committed myogenic precursors. In the adult muscle, in addition to Pax7, large numbers of SCs also express Myf5 (Motohashi and Asakura, 2014) and it has been demonstrated that adult SCs derive from progenitors that first express the myogenic determination gene Myf5 during foetal stages of myogenesis (Biressi et al., 2013). In addition, the role of Myf5 in SC function has been further analyzed by using different mouse mutant strains. Therefore, others Myf5^Cre/Wt^/Pitx2^−/−^ mutant mouse lines showed a small decrease in the number of muscle SCs but within the range of physiological variations and those mutants display significant delay in the regeneration after injury (Ustanina et al., 2007). Crucially, adult Myf5^nlacZ/loxp^ null mice also exhibit perturbed muscle regeneration with a significant increase in muscle fiber hypertrophy, delayed differentiation, adipocyte accumulation, and fibrosis after freeze-injury with an unaltered SC numbers, but they showed a modest impaired proliferation under some conditions *in vitro* (Gayraud-Morel et al., 2007). In our study, we have detected dramatic defects on muscle regeneration in Myf5^Cre/Wt^/Pitx2^−/−^ conditional mutant mice; these results are in consonance with those previous observations and suggest a requirement of Pitx2 function in Myf5^+^ progenitors for the acquisition of a correct SC function. In parallel, we also saw that the number of SCs is normal in the muscles of Myf5^Cre/Wt^/Pitx2^−/−^ mutant but they display a high *Pax7* expression. How the uncommitted character, or the “stemness,” of the embryonic founder cells is retained in SCs remains a matter of ongoing investigation. Importantly, it has been demonstrated that Pax7nGFP high SCs undergo a slow proliferative rate and lower contribution to form muscle precursors during muscle regeneration (Rocheteau et al., 2012). This finding together with the fact that mutant muscle exhibited a reduced number of MYF5^+^ committed SCs and an increase of miR-106b^+^ cells, previously shown as more stemness SC population, could explain the failure in their regenerative capability and emphasize a role for Pitx2 defining specific SC progenitors.

## Data Availability

The original contributions presented in the study are included in the article/Supplementary Material, further inquiries can be directed to the corresponding author.

## Supplementary Material

Supplementary Figure S1
**(A)** Immunoassaying for Pitx2, Pax3 and Myf5 in the DMT at E10.5. **(B)** qRT-PCR analyses for *Pitx2* expression of E10.5 and E18.5 Pax3^Cre/Wt^/Pitx2^-/l-^ mutants embryos in the dermomyotome and limbs. **(C)** Representative images showing the presence of DORV (arrow) Pax3^Cre/Wt^/Pitx2−^/^− embryo at E12.5. ***p<0.001 (*n*= at least 3 embryos per condition).

Supplementary Figure S2
**(A)** Pitx2 expression levels of Pitx2c in Pax3^Cre/Wt^/Pitx2^Wt/-^ heterozygote mice. **(B)** Pitx2 expression levels in the TA muscles of Myf5^Cre/Wt^/Pitx2−^/^− mutant mice. ***p* < 0.01, ****p* < 0.001 (*n* = at least 3 mice per condition).
