# Supplementary material for: Pitx2 Differentially Regulates the Distinct Phases of Myogenic Program and Delineates Satellite Cell Lineages During Muscle Development
Source: Front Cell Dev Biol. 2022 Jul 6;10:940622. doi: 10.3389/fcell.2022.940622 (PMC9298408; doi:10.3389/fcell.2022.940622)
Supplement: Supplementary file 1 [file Table1.DOCX]

**Supplementary Table 1: Primary and secondary antibodies.**

| **Reagent/Resource** | **Reference of Source** | **Identifier** |
| --- | --- | --- |
| Mouse Anti-Desmin | Sigma-Aldrich | D1033 |
| Rabbit Anti-Ki67 (SP6) | Abcam | ab16667  RRID: AB_302459 |
| Mouse Anti-MF20 | Developmental Studies Hybridoma Bank (DSHB) | RRID: AB_2147781 |
| Mouse Anti-Met | Thermo-Fisher | 3D4 |
| Rabbit Anti-Laminin | Sigma-Aldrich | L9393; RRID: AB_477163 |
| Mouse Anti-Pax7 | Developmental Studies Hybridoma Bank (DSHB) | RRID: AB_528428 |
| Mouse Anti-MyoD1 (5.8A) | DAKO | M3512;  RRID: AB_2148874 |
| Mouse Anti-eMyHC (F1.652) | Developmental Studies Hybridoma Bank (DSHB) | RRID: AB528358 |
| Mouse Anti-Myog (FD5) | Developmental Studies Hybridoma Bank (DSHB) | RRID: AB_2146602 |
| Polyclonal goat-anti Pitx2-C16 | (Santa Cruz Biotechnology) | sc-8748 |
| AlexaFluor^®^ Goat Anti-mouse 488 | Thermo Fisher Scientific | A-11001; RRID: AB_2534069 |
| AlexaFluor^®^ Goat Anti-mouse 546 | Thermo Fisher Scientific | A-11030  RRID: AB_144695 |
| AlexaFluor^®^ Goat Anti-rabbit 488 | Thermo Fisher Scientific | A-11008  RRID: AB_143165 |
| AlexaFluor^®^ Goat Anti-rabbit 546 | Thermo Fisher Scientific | A-11035; RRID: AB_2534093 |
| DAPI | Thermo Fisher Scientific | D1306; RRID: AB_2629482 |
