# Supplementary figures and images for: Pitx2 Differentially Regulates the Distinct Phases of Myogenic Program and Delineates Satellite Cell Lineages During Muscle Development
Source: Front Cell Dev Biol. 2022 Jul 6;10:940622. doi: 10.3389/fcell.2022.940622 (PMC9298408; doi:10.3389/fcell.2022.940622)

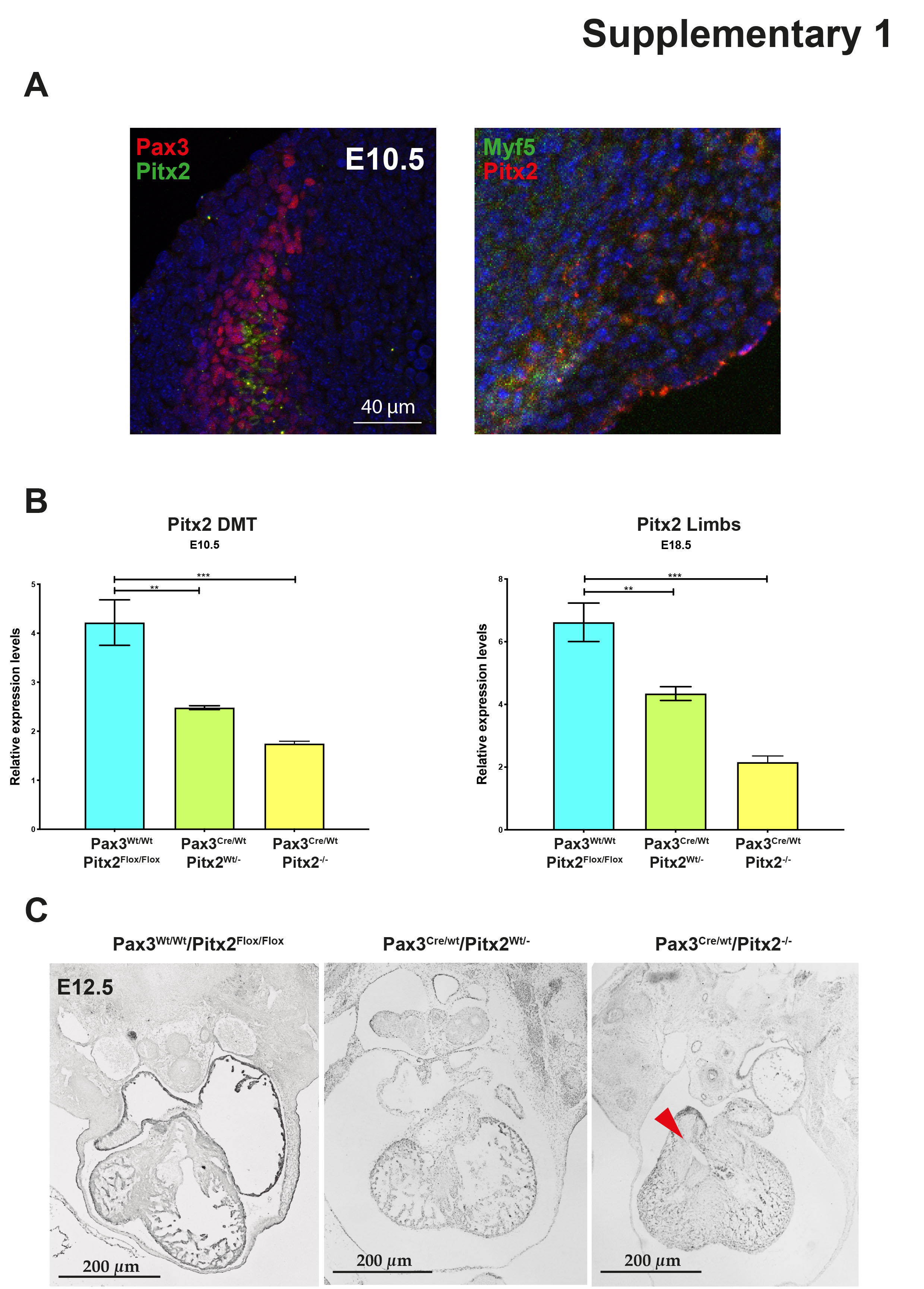

Supplement: Supplementary file 2 [file Image1.jpeg]

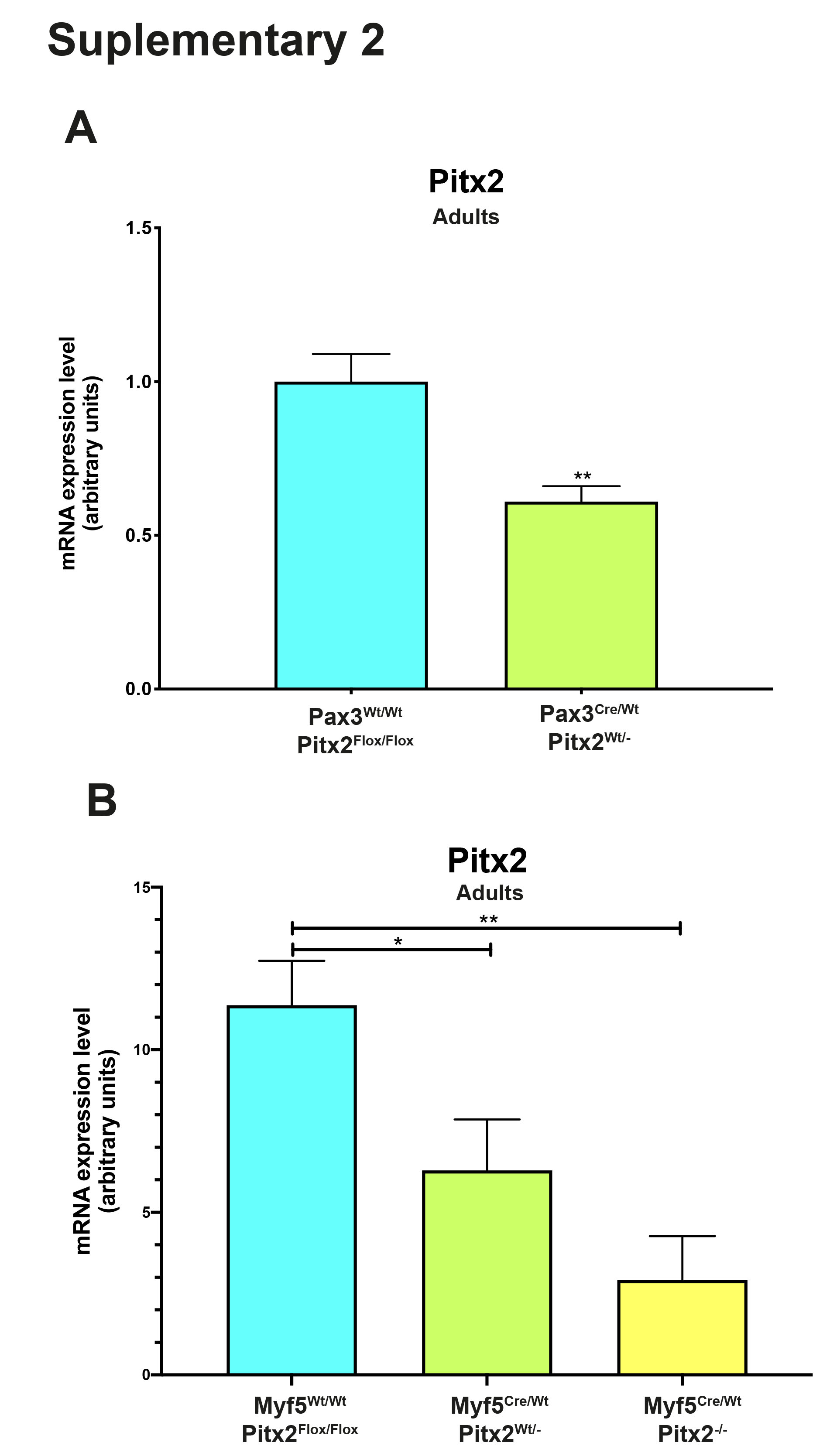

Supplement: Supplementary file 3 [file Image2.jpeg]
